# Clams or Children—Which?

**Published:** 1910-12

**Authors:** 


### Abstracts and Selections.

### Clams or Children—Which?

Do we love clams more than children? If we do not, why did Congress, without hesitation, appropriate $20,000 to pay experts to study clams, and almost unanimously turn down an appropriation of $3000 to pay an expert to study children?

The clam appropriation was passed in 1907, and early in 1908 the child appropriation was killed. In 1905, the State Board of Health presented to the Indiana General Assembly a new health law, which had for its object the conservation of human life. It seems to the board that our State would do well to catch up with other States, and do like sensible, practical work along the lines of preventing disease and saving lives. When the bill was up, a member arose and said: *"I have been requested by my constituents over and over to vote for measures to protect hogs from cholera and trees from scale, but I have never been asked to vote for a measure to protect women and children from preventable diseases."* Of course, the bill was defeated as a crank bill. Had the bill been for hogs, or clams, it would not have been cranky. The reason why the $20,000 clam appropriation passed like greased lightning was because the pearl button makers want clam shells to make pearl buttons from. We can not make anything but men and women out of children; therefore, Congress would not give any money for such an end. It is to be said that Secretary Garfield heartily indorsed the child appropriation, and when he made his argument before the congressional committee a mighty member from Sink-hole arose and asked: "Does this not approach dangerously near the line of encroachment upon the rights of States and municipalities?" When will some people quit thinking it is cranky to protect the human family against disease? No one thinks it is cranky to protect hogs against cholera and trees against scale.—*Bulletin Indiana State Board of Health, Dr. J. W. Hurty, Secretary,* Indianapolis.

